# Comparison of SYSMEX CN-6000 and STAGO STA-R: Effects of hemolysis and lipemia

**DOI:** 10.5937/jomb0-56377

**Published:** 2025-08-21

**Authors:** Seren Orhan, Elif İşbilen

**Affiliations:** 1 Gaziantep University, Faculty of Medicine, Department of Medical Biochemistry, Gaziantep, Turkey

**Keywords:** blood coagulation tests, hemolysis, lipemia, interference, SYSMEX CN-6000, STAGO STA-R, testovi koagulacije krvi, hemoliza, lipemija, interferencija, SYSM EX CN-6000, STAGO STA-R

## Abstract

**Background:**

Routine coagulation testing is critical in diagnosing hemostatic disorders and monitoring anticoagulant therapy. The SYSMEX CN-6000 and STAGO STA-R analyzers utilise different clot detection methods, which may impact test results. This study evaluates the analytical performance of these two automated coagulation analysers and examines the effects of hemolysis and lipemia on routine coagulation tests.

**Methods:**

Blood samples were collected from patients at Gaziantep University Şahinbey Research and Application Hospital and analysed for activated partial thromboplastin time (APTT), prothrombin time (PT), fibrinogen (FBG), and D-dimer using both analysers. Precision, method comparison, and interference studies were conducted following CLSI guidelines. Hemolysis and lipemia were induced in vitro, and their effects on test results were evaluated based on Fraser's criteria.

**Results:**

All precision study CV values were within the acceptable limits of biological variation. APTT results exhibited a significant systematic difference between analysers (r= 0.872), whereas PT (INR), FBG, and D-dimer showed strong correlations (r> 0.945). Hemolysis had a minimal impact at lower concentrations (<1 g/L). However, at 4 g/L, PT bias increased to 2.8% for the CN-6000 and 2.0% for the STA-R, with similar increases observed in APTT and FBG. Lipemia significantly affected CN-6000, which failed to produce PT results at triglyceride levels 14 mmol/L and APTT/FBG results at 28 mmol/L. In contrast, STA-R provided results with biases below 7.3% at all lipemia levels.

**Conclusions:**

Both analysers demonstrated strong analytical performance, though methodological differences influenced APTT measurements. Hemolysis had a minor impact within Fraser's acceptable bias limits, whereas CN-6000 exhibited performance limitations in severely lipemic samples, necessitating preanalytical lipid-reducing strategies. These findings underscore the need for expanded reference range studies and optimised laboratory workflows to enhance coagulation testing reliability.

## Abstract (sr)

**Uvod:**

Rutinsko ispitivanje koagulacije kljucno je za dijagnostikovanje hemostatskih poremećaja i praćenje terapije antikoagulansima. Analizatori SYSM EX c N-6000 i STAGO STA-R koriste različite metode detekcije zgrušavanja, što može uticati na rezultate testova. Ova studija procenjuje analitičke performanse ova dva automatska analizatora koagulacije i ispituje efekte hemolize i lipemije na rutinske koagulacione testove.

**Metode:**

Uzorci krvi su prikupljeni od pacijenata na Univerzitetu Gaziantep, Istraživačka i primenjena bolnica Çahinbey, i analizirani su za aktivirano parcijalno tromboplastinsko vreme (APTT), protrombinsko vreme (PT), fibrinogen (FBG) i D-dimer koristeći oba analizatora. Studije preciznosti, poređenja metoda i interference sprovedene su u skladu sa smernicama CLSI. Hemoliza i lipemija su indukovane in vitro, a njihovi efekti na rezultate testova procenjeni su prema Frejzerovim kriterijumima.

## Introduction

The clinical hemostasis laboratory is a complex environment that employs multiple coagulation assays and various testing methodologies [1]. Routine coagulation tests are essential for screening and diagnosing hemostatic disorders, adjusting anticoagulant dosages, and monitoring therapy. Laboratory results are crucial for clinical decision-making and patient management [2]. STAGO STA-R (Diagnostica Stago, France) is a fully automatic coagulation analyser capable of performing coagulation, chromogenic and immunoturbidimetric tests. The device's working principle is a method that detects changes in the ball's movement in the measuring cuvette when clotting occurs [3] [4]. The SYSMEX CN-6000 (Sysmex Corp. Japan) analyser is a fully automatic coagulation analyser that uses coagulation, chromogenic, and immuno-turbidimetric methods according to the test group. This study aims to evaluate the SYSMEX CN-6000 and STAGO STA-R analysers by assessing precision, method comparison, and interference testing. It focuses on hemolysis and lipemia interferences across a wide concentration range to understand their performance in routine clinical practice.

## Materials and methods

### Sample collection and processing

This study collected blood samples from patients at Gaziantep University §ahinbey Research and Application Hospital during January and February 2022. Samples were randomly selected from residual specimens generated during routine clinical workflow, with no additional blood drawn. Collection and processing were performed following the CLSI H21-A5 guidelines [5]. Blood was collected in tubes containing 3.2% (0.109 mol/L) citrate anticoagulant at a ratio of 1:9 (citrate:blood) using BD Vacutainer Plus tubes (USA) [5]. Plasma was separated by centrifugation at 3000 X g for 10 minutes [6]. All specimens were analysed on both the STA-R and CN-6000 within 4 hours of collection. The commercial kits, controls, and calibrators used in the study are detailed in Table 1. Samples with HIL flags, and insufficient samples were excluded, with HIL flags being assessed on the CN-6000 and STA-R.

**Table 1:** The kits used in the study included control materials and calibrators.

	STAGO STA-R Reagents, Control Materials, Calibrator	SYSMEX CN-6000 Reagents, Control Materials, Calibrator
PT(INR)	STA-Neoptimal ISI : 1.01<br>STA-Coag (Control N and Control P)<br>Pre-calibrated	Thromborel S ISI: 1.05<br>(Control N and Control P),<br>PT Multi Calibrator
APTT	STA-Cephascreen<br>STA-Coag (Control N and Control P)	Dade Actin FS<br>(Control N and Dade Ci-trol 2)
FBG	STA-Liquid Fib<br>STA-Coag (Control N and Control P)<br>STA-Unicalibrator	Dade Thrombin<br>(Control N and Control P), Standard Human<br>Plasma
D-Dimer	STA-Liatest D-DI Plus<br>STA-Liatest (Control N and Control P)<br>Pre-calibrated	Innovance<br>(Control 1 and Control 2), Innovance D-Dimer<br>Calibrant

### Assay principles

The STA-R uses electromechanical clot detection, while the CN-6000 uses an optical technique for clot detection [4] [7]. The APTT, PT, and FBG tests are performed using clotting methods, while the D-dimer test is based on the immunoturbidimetric method. The assays are conducted as follows:

- APTT Test: Citrated plasma is mixed with a contact activator and phospholipids and incubated at 37°C. After incubation, a calcium chloride (CaCI_2_) solution (0.025 mol/L) is added, and the time until clot formation is recorded. The contact activator differs between reagents (e.g., ellagic acid in Dade Actin FS versus factor XII activator in STA Cephascreen).
- PT Test: Thromboplastin and a calcium ion source are added to the plasma at 37°C, and the clotting time is compared to a normal standard.
- FBG Test: The Clauss method measures fibrinogen by recording the time required to form a fibrin clot after adding high concentrations of purified thrombin.
- D-dimer Test: This assay measures an increase in turbidity resulting from the formation of aggregates between D-dimer and monoclonal antibodies.

In this study, activated partial thromboplastin time (APTT), prothrombin time (PT), Fibrinogen (FBG) and D-Dimer results of both analysers were compared. This study compares the analytical performances of STA-R and CN-6000 coagulation analysers using different methodologies for routine coagulation tests. Hemolysis and lipemia (HL) interference is a significant source of analytical error for routine coagulation tests, leading to misinterpretation of results. We also examined the effects of hemolysis and lipemia on PT (INR), APTT and FBG analyses on both analysers.

### Precision study

Precision studies were performed following the CLSI EP15-A3 guidelines [8]. Commercial quality control materials (normal and pathological) were measured at two concentration levels five times per day over five days (25 replicates total). Repeatability and within-laboratory coefficients of variation (CV) were calculated using one-way ANOVA and compared with between-subject biological variation criteria for hemostasis tests. Bias was calculated by comparing the mean control values with the manufacturer's target values using the formula: [(control mean — control target value) / control target value] x 100.

Additionally, all parameters were evaluated within the scope of the RIQAS Coagulation External Quality Assessment Program (2022) and were found to be within ±2 standard deviations of the mean. The acceptable deviation limits set by RIQAS are 14.5% for PT (INR), 15.2% for APTT, 17.9% for fibrinogen (FBG), and 46.6% for D-dimer. The results obtained in this study fully comply with all acceptability criteria, confirming the reliability and accuracy of the coagulation testing.

### Comparison study

For method comparison, at least 88 samples per test were analysed over a wide measurement range. Relationships between methods were evaluated using regression and Bland-Altman plots. Linear regression was applied when the correlation coefficient exceeded 0.975; otherwise, Passing-Bablok regression was used. In Bland-Altman plots, the average of the two methods was plotted on the x-axis and the percentage difference on the y-axis. These percentage differences were compared with between-subject biological variation limits (values obtained from the Westgard database) [9].

### Interference study

The interference study was performed according to the CLSI EP07-ED5 document [10]. Four samples with the same values were obtained by pooling citrated plasma from different patients for hemolysis interference. One sample remained untreated, while the others were added with increasing concentrations of hemolysate. Whole blood anticoagulation with K2 EDTA was centrifuged at SOOOxg for 10 minutes for hemolysate preparation and separated plasma. Then, red cells were washed by adding 0.9% NaCI and centrifuged, and the supernatant was discarded. This process was repeated 5 times. Hemolysis was then induced by adding Triton X-100 (Sigma-Aldrich) to the washed red blood cells. Four groups were created with increasing haemoglobin concentrations: <0.5 g/L (no hemolysis), 0.5-1 g/L ( + ), 1-5 g/L (++), and >5 g/L (+ + +) [11]. Samples were measured on STA-R and CN-6000 instruments for APTT, PT (INR) and FBG [11] [12]. The haemoglobin concentration in the samples was determined using a SYSMEX XN-6000 (Sysmex, Corp. Japan) instrument for the level of hemolysis. Six samples with the same values were aliquoted for the lipemia test, and one was left untreated while the others were added to a commercial intralipid (Clinolipid 20%, Baxter, USA). Triglyceride levels were measured using a Beckman AU 5800 (Beckman Coulter, USA), and APTT, PT (INR), and FBG tests were subsequently performed. Plasma triglyceride concentrations ranged from 0 to 28 mean, standard deviation (SD) and CV values were calculated. In addition, % bias values were calculated by comparing the values obtained in the interference study with the average value of unprocessed samples. Interference from hemolysis and lipemia was compared with the Fraser criteria [13]. Interference effects were compared with Fraser's criteria using the formula 1.96 x (CVa^2^ + CVi^2^) [14], where CVa represents the imprecision of the analytical method and CVi the intra-subject biological variation (values obtained from the Westgard database) [9]. The maximum acceptable interference limit recommended by Fraser according to the formula: 8.8% for PT (CVa 2%, CVi 4%), 6.1% for APTT (CVa 1.4%, CVi 2.7%), 25.4% for FBG (CVa 5.4%, CVi 10.7%).

### Ethics

The study was conducted following the Declaration of Helsinki and was approved by the Human Research Ethics Committee of Gaziantep University (Decision no. 2022/120, Date: 12/10/ 2022). The authors state no conflict of interest.

### Statistics

Statistical analyses were conducted using SPSS version 22.0, a trial version of MedCalc software, and Analyse-it version 2.07 (Analyse-it Software Ltd.). Precision analysis was performed using a one-way analysis of variance (ANOVA) test, with statistical significance set at P<0.05. Normality was assessed using the Shapiro-Wilk test. Descriptive statistics were presented as mean and median values. Pearson's correlation coefficient was used for parametric tests, while Spearman's correlation coefficient was applied for non-parametric tests.

## Results

Precision studies for PT, APTT, FBG, and D-dimer tests using commercially available lyophilised control materials (normal [N] and pathological [P]) are presented in Table 2. All % CV values were within the between-subject biological variation values. Precision study and values are shown in Table 2.

**Table 2:** Precision study of PT(INR), APTT, D-Dimer and FBG tests. N = Normal control, P = Pathological control. CV% = Coefficient of Variation. Acceptable CV values are based on biological variation percentages obtained from the Westgard database.

		SYSMEX CN-6000<br>PT (%)		STAGO STA-R<br>PT (%)		SYSMEX CN-6000<br>APTT (s)		STAGO STA-R<br>APTT (s)	
		N<br>33.7-50.5	P<br>78.6-118.0	N<br>32-46	P<br>72-106	N<br>21.2-28.6	P<br>41.3-52.5	N<br>27-38.5	P<br>44-62
Repeatability	Mean	39.4	95.8	38.6	88.2	24.72	47.01	35.3	54.19
	SD	0.33	0.72	0.6	0.7	0.15	0.45	0.56	0.50
	CV%	0.8	0.7	1.6	0.8	0.6	0.9	1.6	0.9
Within-laboratory	SD	0.33	0.87	0.6	0.7	0.15	0.51	0.64	0.96
	CV%	0.8	0.9	1.6	0.8	0.6	1.1	1.8	1.8
Bias %		6	2.5	1	0.8	0.8	0.2	7	2
Biological<br>Variation %		6.8	6.8	6.8	6.8	8.6	8.6	8.6	8.6
		SYSMEX CN-6000<br>Fibrinogen (mg/dL)		SYSMEX CN-6000<br>Fibrinogen (mg/dL)		SYSMEX CN-6000 D-Dimer (mg/L)		STAGO STA-R<br>D-Dimer (mg/L)	
		N<br>47-127	P<br>198-298	N<br>90-150	P<br>220-335	N<br>0.25-0.37	P<br>1.98-2.96	N<br>0.1-0.5	P<br>1.7-2.5
Repeatability	Mean	81	248	117	278	0.32	2.57	0.32	2.236
	SD	2.46	4.32	3.2	4.5	0.01	0.06	0.007	0.018
	CV%	3	1.7	2.8	1.6	3.1	2.4	2.2	0.8
Within-laboratory	SD	2.46	4.56	3.6	5	0.01	0.08	0.015	0.055
	CV%	5	1.8	3.1	1.8	3.8	3.2	4.6	2.4
Bias %		6	0	2.4	0.1	3.2	4	6.6	4.7
Biological<br>Variation %		15.8	15.8	15.8	15.8	26.5	26.5	26.5	26.5

**Figure 1:**
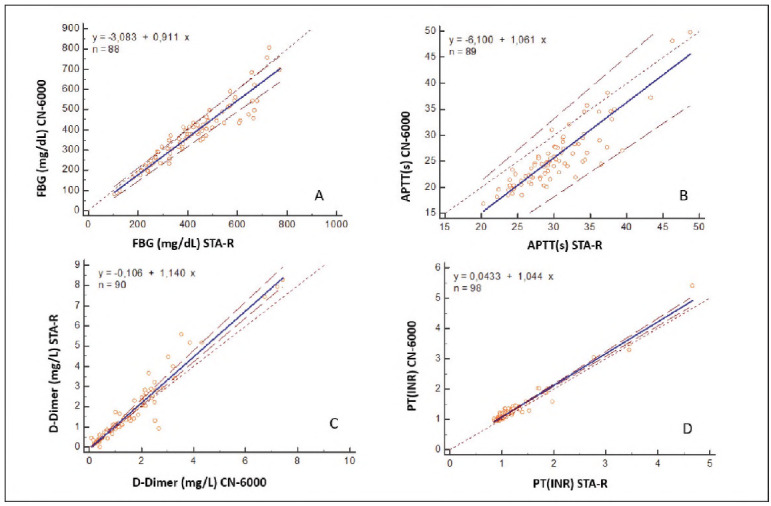
Passing-Bablok regression equations of FBG (A), APTT (B) tests and linear regression equations of D-Dimer (C), PT (INR) (D) tests for STA-R and CN-6000 analyzers. Regression lines are presented with solid blue lines, 95% Cl lines are presented with dashed red lines.

**Figure 2:**
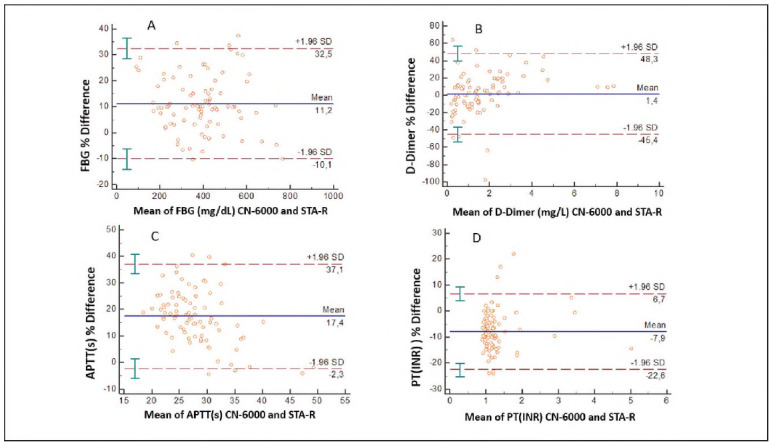
Bland-Altman difference plots for FBG (A), D-Dimer (B), APTT (C), PT (INR) (D) to compare between STA-R and CN-6000 analysers. Showing the % difference between the results on the Bland-Altman chart. The solid line shows the mean of the % difference, the dashed line shows the 95% Cl of this difference.

For the comparison study, 89, 98, 90 and 88 samples were used for APTT, PT (INR), D-Dimer, and FBG tests, respectively. A paired t-test revealed significant differences between the mean values of PT (INR), APTT, D-dimer, and FBG (p<0.05; Table 3). The correlation coefficients of PT (INR), APTT, FBG, and D-Dimer results between the two devices were found to be r=0.982 (p<0,01), r=0.872 (p<0,01), r=0.945 (p<0,01) and r=0.981 (p<0,01) respectively (Table 3). Method comparisons were performed using Passing-Bablok regression analysis. Linear regression was applied for PT (INR) and D-dimer tests (r>0.975), while Passing-Bablok regression was used for APTT and FBG. Regression equations for PT (INR), APTT, D-Dimer and FBG are (y=0.045 + 1.044x, 95% Cl: intercept -0.014 to 0.101 and slope 1.000 to 1.087) (P=0.62), (y=-6.100+1.061x, 95% Cl: intercept -9.995 to -2.662 and slope 0.957 to 1.195) (P = 0.62), (y=-0.106+1.140x, 95% Cl: -0.164 to -0.056 and slope 1.095 to 1.208) (P=0.04) and (y=-5.082+0.911x, 95% Cl: intercept -25.484 to 17.829 and slope 0.856 to 0.968) (P=0.44) respectively (Figure 1A-D). According to Bland-Altman analysis, the % difference between the two methods was -7.9 for the PT (INR) test (95% Cl for the % difference was -22.6 to 6.7), 17.4 for the APTT test (95% Cl for the % difference was -2.5 to 57.1), 1.4 for D-Dimer test (95% Cl for the % difference was -45.4 to 48.5 mg/L), 11.2 for FBG test mg/dL (95% Cl for the % difference was -10.1 to 52.5) (Figure 2A-D). In the in vitro hemolysis interference study, the % bias values of the tests between 0 and 4 g/L haemoglobin concentration were calculated as <5% for all tests in both methods (Table 4). In the in vitro lipemia interference study, CN-6000 could not determine PT (INR) test results at a 14 mmol/L triglyceride concentration. The analyser could not determine values in APTT and FBG tests at a triglyceride concentration of 28 mmol/L. The % bias values of the tests analysed on the STA-R analyser at a triglyceride concentration of 28 mmol/L were PT (INR) 0.9%, APTT 7.5%, and FBG 5.9%. The CN-6000 analyser measured the PT (INR) test in the triglyceride concentration range of 0-8 mmol/L. The highest % bias value in this range was determined as 4.5%. The CN-6000 measured APTT and FBG tests in the triglyceride concentration range of 0-14 mmol/L. In this range, the highest % bias value was determined as 6% for APTT and 5.6% for FBG (Table 4).

**Table 3:** Correlation between CN-6000 and STA-R for coagulation parameter measurements in patient samples.

Parameter	N	CN-6000<br>(Mean ± SD /<br>Median, Range)	STA-R (Mean<br>± SD / Median,<br>Range)	Pearson's r<br>(p-value)	Intercept<br>(95% Cl)	Slope<br>(95% Cl)	Mean %<br>Difference (95%<br>Cl)	Biological<br>Variation<br>(%)
PT (INR)	98	1.19<br>(0.95-5.4)	1.07<br>(0.85-4.67)	0.982<br>(p<0.01)	-0.014 to<br>0.101	1.000 to<br>1.087	-7.9<br>(-22.6 to 6.7)	6.8
APTT (s)	89	26.57±5.8<br>(16.8-137.1)	30.83±5.2<br>(20.3-77.3)	0.872<br>(p<0.01)	-9.993 to<br>-2.662	0.937 to<br>1.195	17.4<br>(-2.3 to 37.1)	8.6
FBG (mg/dL)	88	376±148<br>(81.4-806.8)	418±154<br>(105-772)	0.945<br>(p<0.01)	-25.484 to<br>17.829	0.856 to<br>0.968	11.2<br>(-10.1 to 32.5)	15.8
D-Dimer<br>(mg/L)	90	1.3<br>(0.19-12.38)	1.32<br>(0.09 - 8.93)	0.981<br>(p<0.01)	-0.164 to<br>-0.056	1.093 to<br>1.208	1.4<br>(-45.4 to 48.3)	26.5

**Table 4:** Results of mean+SD and bias (%) on CN 6000 and STA-R for PT, APTT, and fibrinogen according to haemoglobin and triglyceride levels in supernatant.

	Tube	HL<br>Level	PT(INR)<br>CN-6000 Mean<br>±SD, %Bias	PT(INR) STA-R<br>Mean±SD,<br>%Bias	APTT<br>CN-6000 Mean<br>±SD, %Bias	APTT<br>STA-R Mean<br>±SD, %Bias	FBG CN-6000<br>Mean±<br>SD, %Bias	FBG STA-R<br>Mean±SD,<br>%Bias
Hemolysis<br>(g/L)	1	0	1.04+0.007	0.97+0.007	23.2+0.17	27.8+0.09	412+0.7	437+0.7
	2	0,5	1.04+0.01<br>%0.9	0.97+0.007<br>%0.7	23.4+0.2<br>%0.8	27.82+0.09<br>%0.3	411+0.44<br>%0.2	436.8+0.8<br>%0.2
	3	1	1.04+0.01<br>%0.3	0.94+0.007<br>%3.1	23.6+0.13<br>%0.8	28.2+0.23<br>%1.4	411+0.83<br>%0.4	424+0.7<br>%2.9
	4	4	1.07+0.007<br>%2.8	0.95+0.005<br>%2	23.8+0.1<br>%2.5	28.5+0.1<br>%2.5	422 + 1.48<br>%2.6	424+1<br>%2.9
Triglyceride<br>(mmol/L)	1	0	1.09+0.006	1.03+0.007	24.9+0.07	29.39+0.07	276+0.4	285 + 1.09
	2	1,2	1.09+0.008<br>%0.7	1.03+0.01<br>%0.9	24.84+0.08<br>%0.5	29.5+0.11<br>%0.3	276 + 1.03<br>%0.3	284.4 + 1.51<br>%0.7
	3	4	1.11+0.008<br>%1.8	1.02+0.004<br>%0.9	24.5+0.11<br>%1.6	29.3+0.07<br>%0.3	266.8 +<br>1.09 %3.6	276.2 + 1.09<br>%3.1
	4	8	1.14+0.006<br>%4.5	1.02+0.005<br>%0.9	23.9+0.08<br>%4	30.16+0.11<br>%2.6	270.8+1.3<br>%2.1	269 + 1.14<br>%5.6
	5	14	no result	1.02+0.007<br>%0.9	23.4+0.15<br>%6	30.2+0.07<br>%2.7	275.4+4.5<br>%0.2	272.6 + 1.14<br>%4.5
	6	28	no result	1.04+0.007<br>%0.9	no result	31.56+0.11<br>%7.3	no result	268.8+0.8<br>%5.9

## Discussion

This study compared the STAGO STA-R and SYSMEX CN-6000 systems, two new-generation, fully automated coagulation analysers that utilise different detection methods. All parameters demonstrated acceptable repeatability and within-laboratory precision, with coefficients of variation for both normal and abnormal controls remaining below 5% (14). All CV values were within the acceptable limits of between-subject biologic variation criteria for hemostasis tests.

PT (INR) results were consistent between the two methods, as evidenced by the 95% confidence intervals for the intercept and slope (which included 0 and 1, respectively). However, for APTT, the 95% confidence interval for the intercept did not include 0, indicating a significant constant difference between the methods. For FBG, the 95% confidence interval for the slope did not include 1, suggesting a slight proportional difference. A slightly constant and proportional difference was found between the two methods for D-Dimer, as the 95% Cl intercept and slope values in the D-Dimer regression equation did not include 0 and 1, respectively. According to Bland-Altman analysis, the % difference values between the two methods: PT (INR) -7.9 (-22.6-6.7), APTT 18 (2.5-57.1), FBG 11.2 (-10.1-52.5), D-Dimer 1.4 (45.4-48.5). These values are above the biological variation limits (PT; 6.8, APTT; 8.6, FBG; 15.8, D-Dimer; 26.5).

The comparison study indicated that APTT values obtained with the CN-6000 were significantly lower than those from the STA-R, with a weak correlation (r=0.872) between the methods. Notably, 10 samples exhibited APTT values outside the reference range on the CN-6000 while remaining within range on the STA-R. The manufacturer-recommended APTT(Dade Actin FS). Reference ranges (22-28 seconds for the CN-6000 versus 26-57.2 seconds for the STA-R) may contribute to this discrepancy, suggesting that larger reference studies and locally determined ranges could be beneficial. Our findings align with those of Kim et al. (15), who also evaluated the analytical performance of the SYSMEX CN-6000 and STAGO STA-R Max analysers. Their study confirmed the high precision and strong correlation between these systems while highlighting the impact of detection methods on test results. Similar to their findings, our results emphasise that differences in optical and mechanical clot detection techniques can lead to variations in coagulation parameters, reinforcing the need for analyser-specific reference ranges [15]. In a study by Geens T. et al. [7] comparing routine coagulation parameters on the SYSMEX CS5100 analyser and the STAGO STA-R analyser, lower APTT values were observed in the CS5100 compared to the STA-R Evolution analyser.

For FBG and D-dimer, only slight differences were observed, with the CN-6000 recording lower FBG values - possibly due to differing measurement ranges (CN-6000: 180-550 mg/dL; STA-R: 200-400 mg/dL). A moderate correlation was noted between the two analysers for FBG. Geens T. et al. [6,7] found a slight decrease in FBG in the CS5100 compared to the STA-R Evolution device. Before routinely using the CN-6000, information on FBG reference ranges should be provided.

This study evaluated the effects of in vitro-induced hemolysis and lipemia on PT (INR), APTT, and fibrinogen (FBG) tests. These interferences can influence Routine coagulation tests, with the degree of impact depending on the concentration of the interfering substance, the analyser's detection principle, and the reagent used. Hemolysis, caused by biological and analytical factors, may alter the optical properties of a sample, while lipid-induced turbidity can affect photometric tests. Although mechanical clot detection methods are generally less affected by lipemia, high lipid concentrations may still compromise result accuracy [12] [16] [17].

Our findings demonstrated that hemolysis and lipemia had varying effects on coagulation test results. At 4 g/L haemoglobin, PT bias reached 2.8% on CN-6000 and 2% on STA-R, with similar increases observed for APTT and fibrinogen. Mild hemolysis (0.5-1 g/L) had minimal impact, whereas higher levels caused more pronounced deviations.

These results are consistent with previous research, including the study by Kaytaz M. et al. [18], which demonstrated that the CN-5000 and STA-R Max analysers exhibit high precision and minimal interference from hemolysis. Similarly, Nougier et al. [18,19] highlighted that while hemolysis and lipemia can introduce analytical interferences in coagulation testing, the impact is more pronounced when optical detection methods are used. Our findings support these conclusions, reinforcing that mild to moderate hemolysis does not significantly alter PT, APTT, and fibrinogen measurements. However, at higher haemoglobin levels, caution is warranted as more substantial biases may affect diagnostic accuracy [18,19]. In lipemic samples, PT bias at 8 mmol/L triglycerides was 4.5% on CN-6000, whereas STA-R exhibited a lower bias. At 14-28 mmol/L triglycerides, CN-6000 failed to generate results for PT, APTT, and fibrinogen, whereas STA-R continued to provide measurements, with biases remaining below 7.5%. Similarly, Gardnier C. et al. [20], in a study evaluating the CN-6000 hemostasis analyser, reported that while hemolysis did not cause optical interference, optical interference occurred in lipemic samples (triglycerides >10 mmol/L). In the study of Negrini D. et al. [19], in which the lipemia interference of the SYSMEX CS-5100 was examined, it was stated that in highly lipemic samples, the analyser could not determine results for PT, APTT and fibrinogen in lipemic samples. These findings suggest additional lipid-reducing strategies may be necessary when using CN-6000 for highly lipemic samples.

A key limitation of this study is the lack of direct L-index measurements. However, as only HIL-flag-free samples were used and lipemia was induced through controlled lipid emulsions, the variability in lipoprotein composition (e.g., VLDL vs. chylomicrons) was minimised. Therefore, triglyceride concentration was considered a sufficient indicator of lipemia interference under these controlled conditions. The selection of an appropriate interferent for lipemic samples presents a challenge. While patient-derived lipemic samples more accurately reflect pathophysiological lipemia, their heterogeneity makes standardisation difficult. Even when two samples share the same lipemic index or triglyceride concentration, variations in lipoprotein composition can lead to inconsistencies, making replication challenging. Therefore, current recommendations favour standardised lipid solutions with known concentration and composition to ensure reproducibility and reliability in interference studies. Additionally, since hemolysis was induced in vitro, it may not fully replicate the effects of in vivo hemolysis.

Evaluating hemolysis interference in real patient samples would provide a more comprehensive understanding of its clinical impact. Nevertheless, both analysers demonstrated overall reliability, with all bias values remaining within Fraser's acceptable limits, confirming their suitability for routine coagulation testing. Our findings align with those of Gardiner et al. [20], who demonstrated that the SYSMEX CN-6500 analyser maintains good analytical performance while minimising interference from hemolysis and lipemia. The differences observed between the two analysers are likely attributable to reagent variations and methodologies. This study comprehensively evaluates the SYSMEX CN-6000 and STAGO STA-R analysers by integrating precision, method comparison, and interference testing, offering a holistic perspective on their performance in routine clinical practice [21] [22]. A key aspect of this study is the systematic assessment of hemolysis and lipemia interferences across a wide concentration range. This evaluation revealed that the CN-6000 has certain limitations in highly lipemic samples, highlighting the potential need for preanalytical treatment strategies. Additionally, valuable regional data were obtained by conducting this study in a Turkish clinical setting that will provide useful insights for laboratories in similar settings. The findings underscore the importance of enhancing the reliability of coagulation tests and highlight the need for optimised laboratory workflows, providing a potential guideline for improving routine laboratory practices.

## Dodatak

### Acknowledgements

The authors are grateful to the Gaziantep University Research and Training Hospital Hematology Laboratory staff.

### Conflict of interest statement

All the authors declare that they have no conflict of interest in this work.
